# Supplementary material for: The Role of Zinc in Gliotoxin Biosynthesis of Aspergillus fumigatus
Source: Int J Mol Sci. 2019 Dec 8;20(24):6192. doi: 10.3390/ijms20246192 (PMC6940964; doi:10.3390/ijms20246192)
Supplement: Supplementary file 1 [file ijms-20-06192-s001.pdf]

International Journal of
Molecular Sciences

MDPI

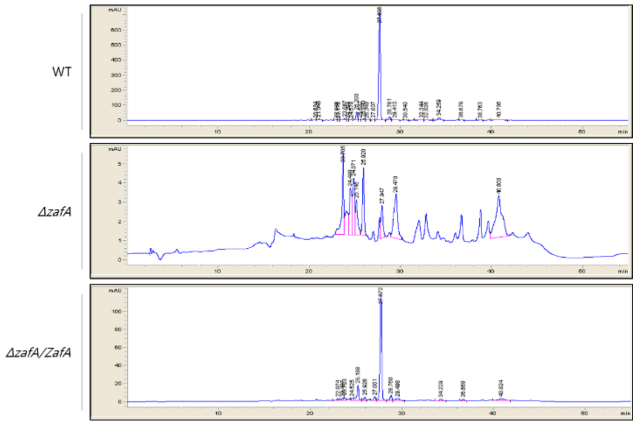

Figure S1. To investigate gliotoxin production in the *ZafA* deletion mutant, the indicated strains were cultured in Czapek-Dox medium for 3 days at 37 °C, and gliotoxin was extracted from the culture medium. The produced gliotoxin was measured with HPLC. RP-HPLC with a UV detector and a polar C18 RP-HPLC column (Agilent Eclipse XDB-C18 (5 μm) 4.6 mm × 250 mm) were used in this HPLC analysis. The flow rate was 1 ml/min, and the mobile phase was methanol:water (50:50).

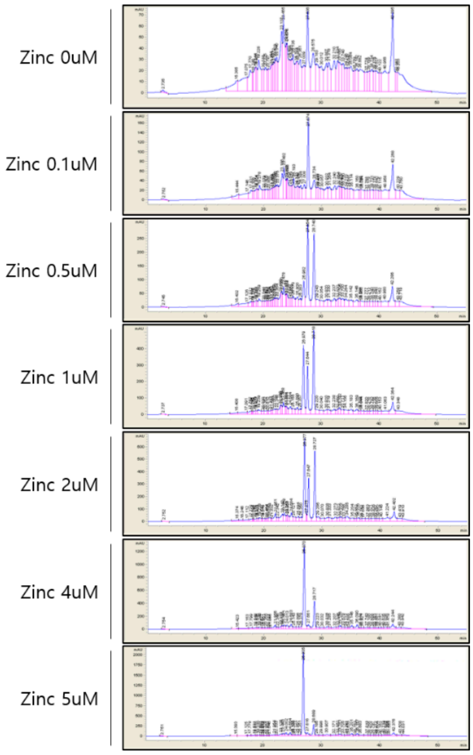

Figure S2. The effect of zinc on gliotoxin production was investigated. Wild-type cells of *A. fumigatus* were cultured in Czapek-Dox medium with the indicated concentration of zinc for 3 days at 37 °C, and gliotoxin was extracted from the culture medium. The produced gliotoxin was measured with HPLC. The analysis condition is as S1.

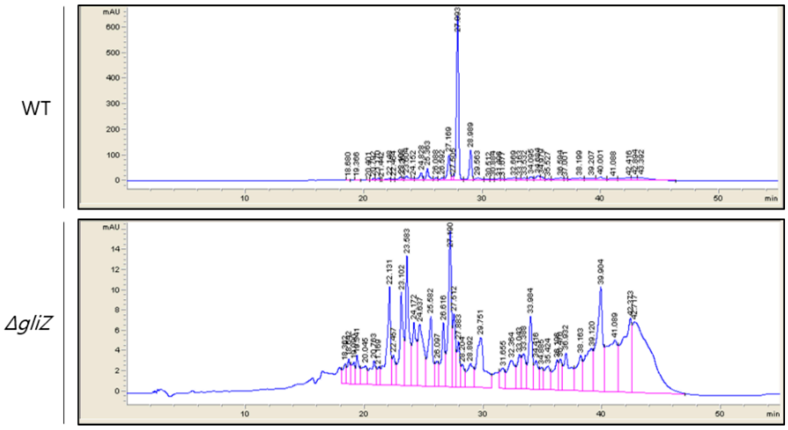

Figure S3. The *GliZ* deletion mutant was used as a control for gliotoxin production to identify the function of GliZ in gliotoxin production. The analysis condition is as S1.

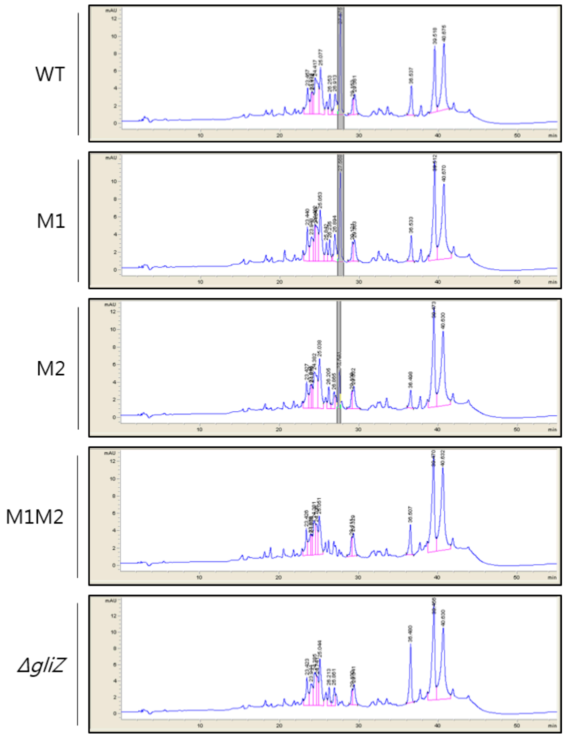

Figure S4. The wild-type and each mutant strain were cultured in Czapek-Dox medium for 3 days at 37°C, and gliotoxin was extracted from the culture medium. The produced gliotoxin was measured with HPLC. The *GliZ* deletion mutant was used as a control. The analysis condition is as S1.

Table S1. List of media and buffer solutions.

Media	Composition	Per liter
AMM	Glucose	10 g
	20X salt mix solution (-$MgSO_4$)	50 ml
	200X $MgSO_4$ solution	5 ml
	1000X Hunter's trace element solution	1 ml
CM	Glucose	10 g
	Yeast extract	1.5 g
	Casamino acid	1.5 g
	100X vitamin solution	10 ml
	20X salt mix solution	50 ml

	200X $MgSO_4$ solution (-$MgSO_4$)	5 ml
	1000X Hunter's TE solution	1 ml
100X vitamin solution	Biotin	0.1 g
	Pyridoxin-HCl	0.1 g
	Thiamin-HCl	0.1 g
	Riboflavin	0.1 g
	p-Aminobenzoic acid	0.1 g
	Nicotinic acid	0.1 g
20X salt mix (-MgSO4)	$NaNO_3$ (sodium nitrate)	120 g
	KCl (potassium chloride)	10.4 g
	KH_2PO_4 (potassium phosphate, monobasic)	16.3 g
	K_2HPO_4 (potassium phosphate, dibasic)	20.9 g
200X MgSO4 solution	$MgSO_4 \cdot 7H_2O$ (magnesium sulfate)	104 g
1000X Hunter's trace element solution	$FeSO_4.7H_2O$ (ferrous sulfate)	5 g
	EDTA	50 g
	ZnSO4.7H2O (zinc sulfate)	22 g
	H3BO4 (boric acid)	11 g
	MnCl2.4H2O (manganous chloride)	5 g
	CoCl2.6H2O (cobaltous chloride)	1.6 g
	CuSO4.5H2O (cupric sulfate)	1.6 g
	(NH4)6Mo7O2.4H2O (ammonium molybdate)	1.1 g

Table S2. List of primers used in this study.

Gene name	Primer name	Primer sequence (5' → 3')
ZafA	Afu.1g10080(ZafA) north_F	AAGATGATTTCTGCCTCGAA
	Afu.1g10080(ZafA) north_R	CAGCATTGAGTCTAGATTGT
GliZ	Afu.6g09630(*GliZ*) north_F	TGCTGCTGCTGCACCCAAGC
	Afu.6g09630(*GliZ*) north_R	CGATGTAGCCGGGAGTGAGG
GliT	Afu.6g09740(*GliT*) north_F	CAGTCGTCTTCGACTCTGGCGTC
	Afu.6g09740(*GliT*) north_R	TTGCGACCGTACCAGCTGTGG
GliN	Afu.6g09720(*GliN*) north_F	AAGACGCCTCGACCCTCCTC
	Afu.6g09720(*GliN*) north_R	TGAGTCGGTACAGCGCCTGC
GliC	Afu.6g09670(*GliC*) north_F	GTTCTTCCGCAACTCGCACC
	Afu.6g09670(*GliC*) north_R	GCTCAGATGAGGCGAGCAGG
GliM	Afu.6g09680(*GliM*) north_F	GCCTGAGGTTCAGTCCTGGCTG
	Afu.6g09680(*GliM*) north_R	AAGGAGACGGACGGCACGAG
GliA	Afu.6g09710(*GliA*) north_F	TCAGTGTCATCATGGCCGGTCTG
	Afu.6g09710(*GliA*) north_R	GTCACGGAGTTTTTGGCGACG
TmtA/GtmA	Afu.2g11120(*GtmA*) north_F	CACCGAAAAGCTCACGGGAC
	Afu.2g11120(*GtmA*) north_R	TTGGAGTCTCCGCTTGGTGG
GliZ	Afu.6g09630(*GliZ*) xho1_F	ctcgagGCGGTTGACTGATATCCCTA
	Afu.6g09630(*GliZ*) hind3_R	aagcttCGCTGACGAGTAGTTTGCTC
	*GliZ*_1st CAAGGT knockout F	CTTTCCCGCCCCGCCTGTCGAGCC
	*GliZ*_1st CAAGGT knockout R	GGCTCGACAGGCGGGGCGGGAAAG
	*GliZ*_2nd CAAGGT knockout F	ACCTCGATCTAACCTCAGCAGGCG
	*GliZ*_2nd CAAGGT knockout R	CGCCTGCTGAGGTTAGATCGAGGT
